# Anterior Spinal Cord Infarction: A Rare Diagnosis With an Uncommon Presentation

**DOI:** 10.7759/cureus.64083

**Published:** 2024-07-08

**Authors:** Sílvia Ferreira, Angelo Fonseca, Filipe Correia, Joana Cunha, Mariana Taveira

**Affiliations:** 1 Intensive Care Unit, Hospital Pedro Hispano, Matosinhos, PRT; 2 Neurology, Hospital Pedro Hispano, Matosinhos, PRT; 3 Internal Medicine, Hospital Pedro Hispano, Matosinhos, PRT

**Keywords:** insulin perfusion, hypertriglyceridemia, chest pain, paraplegia, spinal cord infarction

## Abstract

Spinal cord infarction (SCI) is a rare vascular event accounting for 1% of all strokes. Neurological syndromes may vary according to the arterial territory involved. This condition may differ in onset, severity, and recovery, making it a diagnostic challenge for clinicians. Diagnosis is made on a clinical basis, and neuroimaging (magnetic resonance imaging (MRI)) provides confirmatory evidence.

A 72-year-old male, with a medical history of being overweight, hyperuricemia, dyslipidemia, and cigarette smoking presented to our emergency department (ED) with sudden-onset leg weakness. He reported chest pain with radiation to the back, followed by sudden arm and leg weakness, evolving to inferior limb plegia within four hours. He also noticed a loss of sensation below the breast region. On admission, vital signs were stable. Neurological examination demonstrated paraplegia of inferior limbs with absent deep tendon reflexes. Both pinprick, vibrational, and proprioceptive sensitivities were absent below T6. A diagnostic workup revealed lactescent serum suggesting severe hypertriglyceridemia. A clinical diagnosis of spinal cord infarction was made, which was later confirmed with MRI demonstrating an acute ischemic lesion in the anterior spinal artery (ASA) with the "owl's eye" sign, from T5 with extension to the cone. Neurological examination remained unaltered. He started aspirin and insulin perfusion.

Since spinal cord injury is an uncommon cause of paraplegia, physicians should be extremely cautious. Despite the results of magnetic resonance imaging, the clinical picture was not consistent, which was finally explained by perilesional edema. To our knowledge, this is a rare case combining SCI with hypertriglyceridemia. Notwithstanding the lack of evidence linking reducing triglyceride levels to neurological recovery, insulin infusion was carried out given the hazards associated with sustaining such high levels of triglycerides. We aim to emphasize some characteristic MRI findings and the wealth of possible etiologies contributing to this clinical entity.

## Introduction

Spinal cord infarction (SCI) is a rare vascular event accounting for 1% of all strokes [1,2]. Because the anterior spinal cord anatomy supplies two-thirds of the spinal medulla, damage to this region can have a wide range of physiological effects. SCI can vary in severity, onset, and recovery, which presents a diagnostic difficulty for medical professionals [1].

Diagnosis is made on a clinical basis. However, neuroimaging offers corroborating data that directs medical professionals toward a conclusive diagnosis. Certain distinctive findings are highly useful in differentiating SCI from other myelopathies.

Neurological disorders can differ depending on the artery area affected, even if some patients present with unusual symptoms. There are numerous etiologies that could be causing SCI; however, sometimes, a specific cause is still unknown. Diseases can take many different paths and have varying results. Most patients can recover functionally with the help of intensive rehabilitation programs; however, the prognosis depends in part on the clinical severity at presentation.

With the use of this case study, we hope to raise awareness of the challenges associated with diagnosing SCI in patients who have unusual presentations, as well as the range of etiologies that may be involved. Additionally, we wish to highlight several noteworthy neuroimaging findings as well as the importance of spinal magnetic resonance imaging (MRI) in reaching a conclusive diagnosis.

## Case presentation

**Figure 1:**
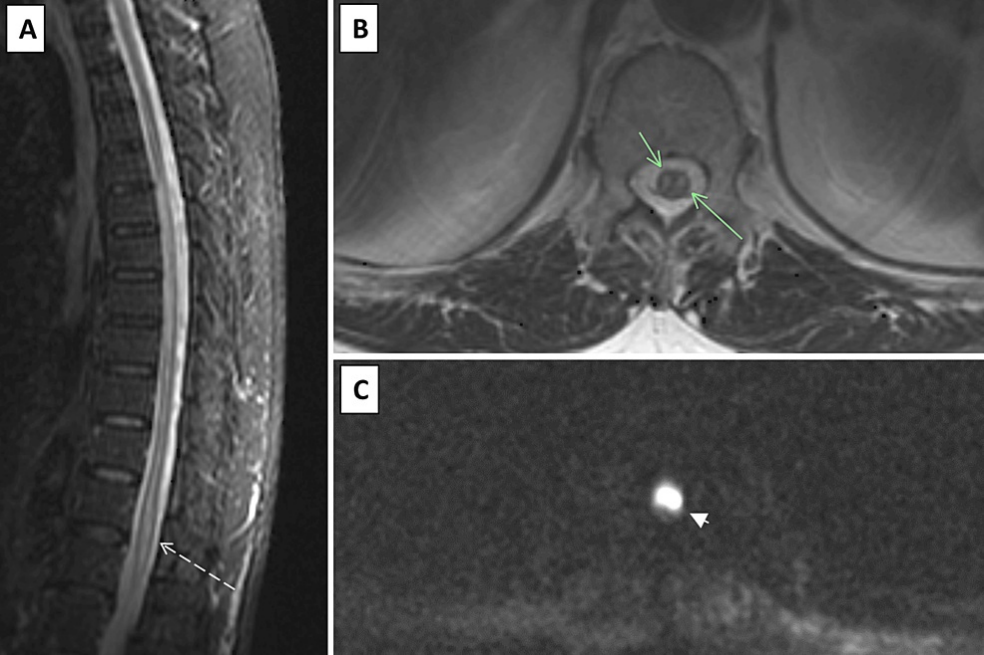
Spinal cord MRI revealed a lesion on the anterior part of the spinal cord below D5, characterized by STIR (dashed arrow) (A), suggestive of acute spinal cord ischemia with associated edema. The presence of a "snake eye" appearance on axial T2 (green arrows) (B) is furthermore observable and its correspondent on DWI (arrowhead) (C), also characteristic of anterior spinal cord ischemia. MRI: magnetic resonance imaging, STIR: short tau inversion recovery, DWI: diffusion-weighted imaging

We present a case of a 72-year-old male with a medical history of being overweight, hyperuricemia, dyslipidemia with severe hypertriglyceridemia (last measurement one year before with 1,058 mg/dL; medicated with ezetimibe 10 mg and atorvastatin 20 mg), and cigarette smoking who presented to our emergency department (ED) with sudden-onset leg weakness. He reported chest pain with radiation to the back, followed by sudden arm and leg weakness, evolving to inferior limb plegia within four hours. He also noticed a loss of sensation below the breast region. On admission, he was apyretic, eupneic, normotensive, and normocardic. Neurological examination demonstrated flaccid paraparesis of inferior limbs with absent deep tendon reflexes. Both pinprick, vibrational, and proprioceptive sensitivities were absent below T6. Upper limb and cranial nerve examination was unremarkable. Five days later, he developed urinary retention and was submitted to vesical catheterization. Posterior monitorization excluded atrial fibrillation. Computed tomography angiography (CTA) excluded aortic dissection. A diagnostic workup revealed lactescent serum suggesting severe hypertriglyceridemia. The coagulation test yielded normal findings (activated partial thromboplastin time (aPTT): 28.4 seconds, prothrombin time (PT): 10.2 seconds). After a spinal cord infarction diagnosis was made clinically, the patient was hospitalized in the intermediate care unit and given supportive care. On day 2, he was submitted to dorsal spine magnetic resonance imaging (MRI) that confirmed the diagnosis, demonstrating an acute ischemic lesion in the anterior spinal artery (ASA) with the "owl's eye" sign on the medulla, from T5 with extension to the conus medullaris (Figure 1A-1C).

Insulin perfusion (8 U/hour) was started as a treatment for hypertriglyceridemia, and aspirin 100 mg was begun as secondary prevention. Insulin infusion was stopped after triglyceride levels dropped below 500 mg/dL, and high-intensity statin and fibrate therapy were started instead. Additional examinations were carried out to look into possible thrombotic reasons. He had recent endoscopic studies without any significant alteration, and an echocardiogram was done, demonstrating good biventricular function, without segmental akinesis. Cerebral MRI revealed periventricular lesions suggestive of atherosclerotic microangiopathy.

At discharge, he maintained paraplegia, but deep tendon reflexes were present (although hardly arousable). He had no anal sensation or muscle tone. He also developed neuropathic pain above a sensitive level, being controlled with gabapentin. After being admitted to the hospital for 20 days, the patient spent two months at a spinal rehabilitation facility. Prior to release, the most recent triglyceride reading was 366 mg/dL.

After that time, he returned home in a wheelchair. He was evaluated as an outpatient five months later, without any functional recovery. Nevertheless, he can move independently in the wheelchair. The patient is able to carry out some activities of daily living such as dressing and eating. He spends most of the day writing, reading, watching television, or chatting on his cell phone.

## Discussion

Spinal cord infarction is an uncommon vascular event with a variety of clinical manifestations and outcomes. The spinal medulla is anatomically restrained to a very confined space. Its irrigation supply is assured by three main vessels: one anterior spinal artery (ASA) and two posterior spinal arteries [3]. ASA is the longest artery in the body, and it is smaller in diameter across the thoracic region, where vascular supply to the spinal medulla receives contribution from radicular arteries. The anterior two-thirds of the spinal cord receives irrigation from it. Spinal irrigation is largely dependent on perfusion pressure, and metabolic demand is more prominent among grey matter at the cervical and lumbar regions [4].

Clinical presentation can be related to the arterial territory affected, and patients usually report chest or back pain before neurological presentation [5], like our patient described in the ED. However, there have been documented cases with unusual presentations [6,7]. Despite only having ASA thrombosis, our patient's anterior and posterior spinal cord condition mimicked the clinical presentation of a complete spinal cord transection. The MRI-reported perilesional edema was the only explanation we could find for this condition, which made the case's presentation unusual.

Although the diagnosis of SCI may be based on clinical findings with a high level of suspicion, MRI provides the definitive diagnosis, showing hyperintensities on T2-weighted sequences [7]. If clinical suspicion is high, a second examination should be performed later in the course of illness, as MRI alterations may not typically manifest in the early hours. MRI also excludes other differential diagnoses of sudden leg paralysis such as compressive myelopathy or transverse myelitis. Hyperintensities documented on T2-weighted sequences are not specific of SCI. A confirmatory sign of SCI is present only in a minority of cases and comprises an "owl eye" or "snake eye" sign [5,8], which was present (Figure 1B, 1C) in our patient's examination. This is a novel observation that manifests as bilateral hyperintense symmetric, circular, or ovoid foci on T2-weighted MR axial sequences in the spinal cord's anterior horn cells [8,9]. Although the exact etiology is unknown, the most widely accepted theory is that it is caused by injury or blockage of the anterior spinal artery. Anatomopathologically speaking, there is cell loss in the anterior horn together with cystic necrosis at the intersection of the posterior ventrolateral column and the central grey matter. Consequently, the appearance of "owl eyes" is highly susceptible to anterior spinal cord infarction [9,10].

A number of etiologies for SCI have been reported; they include cases associated with arterial thrombosis and complications after thoracic aortic surgery, which are reported sometimes. In many cases, no clear etiology is found.

Concerning the case presented here, our patient's medical history included dyslipidemia, smoking, and a history of hyperuricemia, all of which increased his vascular risk [11,12]. Systematic Coronary Risk Evaluation 2-Older Persons (SCORE2-OP)-calculated risk profile revealed 38% of 10-year risk of cardiovascular event. Furthermore, the patient's excessive triglyceride levels at home likely contributed to the development of atherosclerosis [11,12].

The patient's presentation with lactescent serum on admission was concerning since these levels may lead to the development of acute pancreatitis, worsen necrotization, and exacerbate the development of multiple organ failure. We did not find literature establishing the causal link between spinal cord ischemia and hypertriglyceridemia as well as a targeted treatment. However, treatment to lower serum levels has become critical given the hazards associated with sustaining such high levels of triglycerides. Literature has reported the use of insulin perfusion to successfully treat acute pancreatitis in patients with severe hypertriglyceridemia (triglyceride level above 1,000 mg/dL) [13,14]. Insulin infusion was performed despite the lack of evidence that reduced triglyceride levels are associated with neurological improvement. Triglyceride levels were below 500 mg/dL three days later, and insulin perfusion was discontinued. After that, he began taking fibrate and high-intensity statins as recommended by the literature [15].

The prognosis of these patients is variable. Authors consider that advanced age, severe impairment at presentation, and lack of improvement after 24 hours are poor prognostic factors [6,16]. Many patients are able to recover from their deficits, with intensive programs of rehabilitation over time. The case presented here had a poor functional outcome since there was a lack of improvement in the first 24 hours of treatment, and the etiology was not completely clear. The identification and treatment of all possible contributors may prevent future events; nevertheless, we believe that unfortunately, it did not alter the functional outcome of our patient.

## Conclusions

This case report highlights several interesting aspects. On the one hand, it was a severe initial presentation with atypical manifestations. Despite documentation of anterior spinal artery territory infarction, there was a "complete" spinal syndrome. MRI gives physicians important information and aids in ruling out other clinical entities, allowing them to make a conclusive diagnosis. Furthermore, certain imaging indicators, such as "owl eye" or "snake eye," strongly suggest anterior spinal cord infarction. On the other hand, this case emphasizes the wealth of possible etiologies contributing to this clinical entity. This patient's significant hypertriglyceridemia and elevated vascular risk were likely contributing factors to his illness. Hypertriglyceridemia is an independent risk factor and has been rarely linked to this clinical entity. Clinical suspicion must be high in order to establish a prompt diagnosis and provide an early establishment of treatment.
